# Supplementary material for: WordCommentsAnalyzer: A windows software tool for qualitative research
Source: F1000Res. 2018 Sep 4;7:536. Originally published 2018 May 3. [Version 2] doi: 10.12688/f1000research.14819.2 (PMC6137411; doi:10.12688/f1000research.14819.2)
Supplement: Supplementary file 3 [file f1000research-7-17614-s0002.tgz › b2a79496-4ada-47c4-9682-a99ce867836d.zip!/Eight Iranian Health Journals/Yafteh _13.docx]

© Yafteh  . This work is licensed under CC BY 4.0 (https://creativecommons.org/licenses/by/4.0/).

Volume & Issue: دوره 17 - شماره 1

Title: بررسی میزان رعایت منشور حقوق بیمار از دیدگاه بیماران بستری شده در بیمارستان شهدای عشایرخرم آباد در سال 1392

http://yafte.lums.ac.ir/article-1-1841-fa.html

مقدمه: از جمله مهمترین شاخصهای ارزیابی کیفی ارائه خدمات سلامت بررسی وضعیت رعایت حقوق بیمار و نحوه تعامل ارائه دهندگان و گیرندگان خدمات سلامت است. این مطالعه به منظور بررسی رعایت منشور حقوق بیمار از دیدگاه بیماران بستریشده در بیمارستان شهدای عشایر که مرجعیت آن به عنوان یک مرکزآموزشی درمانی جنرال در سطح استان می‌باشد در سال 1392 انجام شده است. بحث و نتیجه‌گیری: در مجموع بر اساس نتایج حاصل از این مطالعه چنین استنباط میشود که بیماران میزان رعایت منشور حقوق خود را در اغلب موارد متوسط می‌دانند ولی باید در جهت بهبود عوامل موثر در آن تلاش کرد. یافته‌ها: نتایج به دست آمده در این مطالعه نشان دادند که میزان رعایت حقوق بیمار (در تمامی ابعاد به صورت کلی) از دیدگاه ۲/۱۱ درصد بیماران خوب، از دیدگاه ۷/۸۶ درصد بیماران متوسط و از دیدگاه ۱/۲ درصد بیماران ضعیف بود. سن بالاتر، تحصیلات پایینتر، سکونت در شهر، مدت بستری کمتر و بستری در بخشهای داخلی و عفونی از عوامل مؤثر بر بهتر بودن دیدگاه بیماران و نیز میزان رعایت منشور حقوق بیمار بودند (05/۰P<). بین جنسیت بیمار و سابقه بستری قبلی با میزان رعایت منشور حقوق بیمار رابطهی معناداری وجود نداشت. مواد و روش‌ها: در این مطالعه مقطعی، ۲۸۶ نفر از بیماران بستری شده در بیمارستان شهدای عشایر در سال 1392 وارد مطالعه شدند و میزان رعایت منشور حقوق بیمار از دیدگاه آنها به وسیله پرسشنامه مورد بررسی قرار گرفت.

Title: بررسی فروانی سل ریوی اسمیر مثبت در زندان

http://yafte.lums.ac.ir/article-1-1842-fa.html

مقدمه: با توجه به اینکه تمام شرایط گسترش سل شامل، ازدحام جمعیت، عفونت HIV، اعتیاد تزریقی، سوء تغذیه و فقر، در زندان فراهم است و از طرفی سل شایعترین علت بستری و مرگ در بیماران HIV و زندانیان را تشکیل می دهد و زندانها مخزن اصلی، انتشار سل بخصوص سل مقاوم به چند دارو به سطح جامعه هستند، لذا نیاز به بررسی جامع در این زمینه وجود دارد. بحث و نتیجه‌گیری: مقایسه مطالعه ما نسبت به اکثر مطالعات در استفاده از الگوریتم مشخص، شاخص WHO، اسکورهای بالینی تکمیلی Total ،BMI و سابقه قبلی درمان سل و همچنین استفاده بیشتر از گرافی قفسه سینه بود، که دقت ما را در اسکرینینگ اولیه و بعدی افزایش داد. بنابراین با بیماریابی فعال بصورت دوره ای و درمان به موقع سل می‌توان بطور زودرس از انتشار گسترده سل در زندان و جامعه ممانعت بعمل آورد و از میلیاردها دلار خسارت جلوگیری نمود. یافته‌ها: از 2200 زندانی، 2030 (27/92%) ویزیت بعمل آمد که 469 نفر (1/23%) معیار WHO (5≤P) ،287 نفر (3/14%) معیار Total (8≤T) و 159 نفر (83/7%) معیار BMI (20B<) را کسب کردند 2 نفرهم دارای سابقه قبلی درمان سل بودند. تعداد کل معیارهای مثبت 915 بود. که با حذف مشترکات612 نفر (14/30%) به مرحله بعد راه یافتند که از این تعداد، از 470 نفر (8/76%) CXR، 198 نفر (35/32%) اسمیر بعمل آمد. در نهایت، که 5 نفر سل ریوی "اسمیر مثبت"، تشخیص داده شد که همگی (100%) 5≤W،4 نفر(80%)، 8≤Total و1 نفر(20%) دارای 20˂BMI، ولی هیچکدام (0%) سابقه قبلی درمان سل نداشتند. مواد و روش‌ها: این مطالعه توصیفی و به صورت سرشماری و در یک زندان کرمانشاه طی سال 86-1385 صورت گرفت. بعد از تمهیدات لازم اقدام به بیماریابی سل با پرسشنامه و معاینه ریه و محاسبه Body Mass Index (BMI) گردید. علائم بالینی سل به دو صورت اسکوربندی شدند، یکی طبق دستور العمل سازمان بهداشت جهانی (score WHO) و دیگر علائم بالینی سل را که در دستورالعمل WHO ذکر نشده بود، جداگانه تحت عنوان Total، اسکور بندی کردیم، در نهایت همه افراد دارای 5 از 7 نمره WHO یا 8 از 14 نمره Total، یا BMI کمتر از20، یا سابقه قبلی درمان سل، تحت غربالگری اولیه و بررسی از نظر سل قرار گرفتند. سرانجام از افراد با تشخیص سل، تستHIV به عمل آمد و داده ها مورد آنالیز آماری قرار گرفتند.

Title: بررسی شدت و عوامل مستعد کننده بیماری کتو اسیدوز دیابتی در بیماران بستری در بیمارستان شهدای عشایر خرم‌آباد

http://yafte.lums.ac.ir/article-1-1843-fa.html

مقدمه: کتواسیدوز دیابتی عارضه حاد دیابت می باشد و ازفوریتهای پزشکی است که معمولاً در بیماران دیابتی نوع I دیده می شود و ممکن است تظاهری از بیماری دیابت تیپ I تشخیص داده نشده مخصوصاً در کودکان باشد و همراه با کمبود انسولین، کاهش حجم و تغییر وضعیت روانی می باشد.هدف از انجام این مطالعه بررسی شدت و عوامل مستعد کننده بیماری کتو اسیدوز دیابتی در بیماران بستری در بیمارستان شهدای عشایر خرم آباد. بحث و نتیجه‌گیری: عدم آگاهی بیماران و خانواده‌های آنان در شناخت علائم حاد بیماری و همچنین عدم پذیرش درمان که منجر به قطع مصرف انسولین می‌گردد علت احتمالی شدید بودن بیماری در افراد مطالعه ما می باشد. یافته‌ها: در بایگانی بیمارستان 38 پرونده از افراد که مبتلا به کتو اسیدوز دیابتی بودند موجود بود. از نظر توزیع جنسی در صد زنان مبتلا به این بیماری بیشتر از مردان بود. از نظر سنی بیشترین فراوانی در گروهای سنی 10-9 سال و 29-20 سال بود. از نظر توزیع فراوانی عوامل مستعد کننده بیشترین عامل منفرد مستعد کننده بیماران عدم مصرف انسولین و قطع صرف انسولین بود. از نظر شدت، بیماری اغلب شدید بود که در زنان بیشتر دیده شده است. مواد و روش‌ها: در این روش که مطالعه اپیدمیولوژیک توصیفی می باشد کلیه بیماران دیابتی که بعلت کتواسیدوز دیابتی در بیمارستان شهدای عشایر از خرداد 88 تا خرداد 92 بستری شده بودند و پرونده های آنها که در بایگانی بیمارستان موجود بود توسط همکار طرح، طبق معیارهای از پیش تعیین شده، اطلاعات آنها استخراج و وارد پرسشنامه شد.

Title: نیازهای آموزشی پزشکان عمومی درباره بیماری بروسلوز (خرم آباد- ایران)

http://yafte.lums.ac.ir/article-1-1844-fa.html

مقدمه: بیماری بروسلوز خسارات اقتصادی، دامپزشکی و بهداشت عمومی قابل توجهی را به اجتماع تحمیل می کند. با توجه به شیوع بالای تب مالت در استان لرستان، این مطالعه با هدف تعیین میزان نیازهای آموزشی پزشکان عمومی در مورد بیماری بروسلوز در شهرستان خرم آباد انجام شد. بحث و نتیجه‌گیری: میزان نیازهای آموزشی اکثر پزشکان عمومی در شهر خرم آباد درباره بیماری بروسلوز در حد متوسط است. با توجه به اهمیت تشخیص و درمان صحیح این بیماری پیشنهاد می‌شود که در برنامه آموزش مدون پزشکان توجه ویژه ای به برنامه های آموزشی مرتبط با بروسلوز معطوف شود. یافته‌ها: یافته ها نشان داد که 6/28 درصد از پزشکان عمومی در مورد بیماری بروسلوز نیاز آموزشی دارند. بیشترین نیاز آموزشی در حیطه تشخیص (6/78 درصد افراد) و کمترین نیاز آموزشی در حیطه پیشگیری (1/17 درصد افراد) بود. نمره نیازسنجی بین مشارکت کنندگان زن و مرد اختلاف آماری معنی دار نداشت اما پزشکان شاغل در مراکز روستایی، نیاز آموزشی بیشتری داشتند. مواد و روش‌ها: در این مطالعه مقطعی ، 140 نفر از پزشکان عمومی شاغل در شهر خرم آباد در سال 1392 به شیوه سرشماری وارد مطالعه شدند. ابزار جمع آوری اطلاعات پرسشنامه ای پژوهشگر ساخته مشتمل بر 42 سوال در چهار حیطه پیشگیری، تشخیص، درمان و پیگیری بروسلوز بود. تجزیه و تحلیلی آماری با استفاده از نرم افزار SPSS نسخه 16 آزمون های آماری پیرسون، کای اسکوئر و من ویتنی انجام شد. مقدار P کمتر از 05/0 از نظر آماری معنی دار در نظر گرفته شد.

Title: بررسی وضعیت باکتری‌ها، قارچ‌ها و بیومس در ریزگرد‌های هوای شهر خرم‌آباد در تابستان و پاییز سال 1391

http://yafte.lums.ac.ir/article-1-1845-fa.html

مقدمه: ریزگردها ترکیبی از آلاینده‌ها در اتمسفر می‌باشند که بخشی از این ذرات را بیوآئروسل‌ها تشکیل می‌دهند. هدف از این مطالعه بررسی وضعیت باکتری‌ها، قارچ‌ها و بیومس در ریزگرد‌های هوای شهر خرم‌آباد بوده است. مواد و روش‌ها: این مطالعه مقطعی در تابستان و پاییز سال 1391 در شهر خرم آباد انجام گرفت. نمونه برداری با استفاده از دستگاه نمونه بردار با حجم بالا انجام گرفت. برای شمارش و شناسایی عوامل قارچی از محیط کشت‌های اختصاصی، برای تعیین باکتری از روش HPC و جهت تعیین بیومس از معیار پروتئین بر اساس روش برادفورد استفاده گردید. همچنین ارتباط این متغیرها با پارامترهای هواشناسی مورد مطالعه قرار گرفت. بحث و نتیجه‌گیری: با افزایشPM10 غلظت بیومس در هوا افزایش یافته، اما تأثیر چندانی بر غلظت باکتری‌ها و قارچ‌ها در هوا نداشت. فاکتورهای هواشناسی مانند دما، رطوبت، سرعت باد، مدت تابش و میزان تشعشع خورشید تأثیر بسزایی در غلظت بیوآئروسل‌ها در هوا داشت. یافته‌ها: بالاترین و پایین‌ترین مقدار PM10 به ترتیب در تیر ماه با غلظتg/m3µ 18/275 و در شهریور ماه با غلظتg/m3µ 45/92 بود. بیشترین میانگین ماهانه تعداد کلنی باکتری و قارچ اندازه گیری شده، به ترتیب 605 و 120 عدد در هر متر مکعب هوا و مربوط به ماه‌های آذر و آبان بود. همچنین بیشترین غلظت میانگین ماهانه پروتئین g/m3µ 30-27، مربوط به ماه‌های مرداد، شهریور و آذر بود. با افزایش غلظت PM10 غلظت بیومس در هوا افزایش معنی‌داری نشان داد.

Title: بررسی علل گرایش به مواد مخدر از نظر بیماران با تشخیص مسمومیت مواد مخدر و اعتیاد مراجعه کننده به بیمارستان‌های آموزشی شهر خرم‌آباد

http://yafte.lums.ac.ir/article-1-1846-fa.html

مقدمه: کمتر پدیده ای را می‌توان یافت که همانند اعتیاد جوامع بشری را مورد تهدید قرار داده باشد. با وجود خطرات و عوارض ناشی از اعتیاد هر روزه به شمار قربانیان این دام مرگبار افزوده می‌شود. لذا برآن شدیم که به بررسی علل گرایش به مواد مخدر از نظر بیماران مراجعه کننده با تشخیص مسمومیت مواد مخدر و اعتیاد در بیمارستان‌های آموزشی شهر خرم آباد بپردازیم. بحث و نتیجه‌گیری: عوامل فردی، خانوادگی و اجتماعی اقتصادی به صورت هماهنگ بستر گرایش به سمت مواد مخدر را فراهم می‌کنند و امید است که نتایج این مطالعه موجبات افزایش آگاهی برنامه ریزان در اجرای سیاست‌ها و اقدامات پیشگیرانه و کنترل کننده اعتیاد جهت جلوگیری از پیامدهای آن را فراهم ‌نماید. یافته‌ها: 11 بیمار مؤنث و 89 بیمار مذکر مورد بررسی قرار گرفتند. بیش‌ترین فراوانی در گروه سنی 39-30 سال قرار داشت. 81% بیماران شهری و 19% روستایی بودند. کسب لذت و افزایش توان جسمی برای کار، رهایی از مشکلات زندگی، تسکین ناراحتی‌های روحی، وجود افراد معتاد در خانواده، همرنگ شدن با دوستان ناباب و همچنین بیکاری و ارزان و در دسترس بودن مواد مخدر از شایع‌ترین علل گرایش به مصرف مواد بود. مواد و روش‌ها: مطالعه در قالب اپیدمیولوژیک توصیفی بر روی 100 نفر، تحت رضایت آگاهانه در سال 1393 انجام شد. ابزار پژوهش یک پرسشنامه‌ دو قسمتی بود که قسمت اول در زمینه خصوصیات فردی و قسمت دوم حاوی سؤالاتی در زمینه عوامل شغلی، اقتصادی، تحصیلی، خانوادگی و عوامل اجتماعی گرایش به مواد مخدر بود. پس از گردآوری داده‌ها، بررسی و تحلیل آماری انجام شد.

Title: بررسی ارتباط بین خواب آلودگی با پارامترهای پلی سومنوگرافی در مبتلایان اختلال تنفسی حین خواب مراجعه کننده به درمانگاه فوق تخصصی بقیه الله شهر قم 1391

http://yafte.lums.ac.ir/article-1-1847-fa.html

بحث و نتیجه‌گیری: بر اساس داده های موجود، مهمترین پارامتر در بروز خواب آلودگی در بیماران مبتلا به اختلالات تنفسی حین خواب میزان اندکس بیداری و متوسط اشباع اکسیژن شریانی می باشد. مواد و روش‌ها: با لحاظ معیارهای ورودی و خروجی، 112 بیمار مراجعه کننده به کلینیک خواب درمانگاه فوق تخصصی بقیه الله شهر قم در سال 1391 در مطالعه تحت مطالعه قرار گرفتند. از بیماران اطلاعات دموگرافیک و پرسشنامه خواب آلودگی گرفته شد. مقایسه بین خواب آلودگی با پارامترهای پلی سومنوگرافی و آنتروپومتریک انجام شد. داده ها توسط نرم افزار SPSS نسخه 18 توسط شاخصهای آمار مرکزی و نیز توزیع فروانی و همچنین توسط آزمون های T مستقل، ضریب همبستگی پیرسون و نیز کای اسکوئر در سطح معنی داری 05/0>P تجزیه و تحلیل شد. یافته‌ها: سن متوسط بیماران 07/55 سال و 09/58% آنها مرد بودند. شاخص توده بدنی 34/34 کیلو گرم بر مجذور مترمربع، متوسظ اندکس آپنه و هیپوپنه 62/43، متوسط اسکور خواب آلودگی بالای ده 83/12، و متوسط اندکس بیداری 64/30 بود. ارتباط قوی بین اسکور خواب آلودگی بالای 10 با اندکس بیداری (009/0=P) و متوسط اشباع اکسیژن شریانی (029/0=P) مشاهده شد. اما میان خواب آلودگی با پارامترهای دیگر پلی سومنوگرافی ارتباط معناداری دیده نشد (005/0˂P). مقدمه: به خواب آلودگی که در زمان نامناسب حادث شود و یا عملکرد روزانه فرد را مختل کند، خواب آلودگی مفرط طی روز گویند. در این بین اختلال تنفسی حین خواب یکی از شایعترین علل خواب آلودگی می باشد. هدف از این مطالعه یافتن مهمترین پارامترهایی است که در پلی سومنوگرافی بیماران اختلالات تنفسی حین خواب با خواب آلودگی مرتبط هستند.

Title: بررسی تأثیر ماساژ پا بر کیفیت خواب شبانه بیماران همودیالیزی

http://yafte.lums.ac.ir/article-1-1848-fa.html

مقدمه: مطالعات نشان داده اند، حدود 20 تا 80 درصد بیماران همودیالیزی، دارای مشکلات خواب هستند که می تواند منجر به کاهش کیفیت زندگی آنان شود. با توجه به آمار بالای بیماران همودیالیزی در عصر حاضر، ارتقاء سطح سلامت آنان از مسائل مهم بهداشتی تلقی می گردد. در این راستا پژوهش حاضر، با هدف تعیین تأثیر ماساژ پا بر کیفیت خواب شبانه بیماران همودیالیزی انجام گرفت. بحث و نتیجه‌گیری: با توجه به نتایج پژوهش، ماساژ پا بر کیفیت خواب بیماران تحت همودیالیز تأثیر مطلوبی دارد و می‌تواند به عنوان یک روش مفید در بهبود کیفیت خواب بیماران در بخش های همودیالیز، اجرا و آموزش داده شود. یافته‌ها: تغییرات ایجاد شده در نمره پیتزبرگ نشان داد که، ماساژ پا در گروه آزمون در کیفیت خواب موثر بوده و با استفاده از آزمون آماری فریدمن مشخص شد مدت خواب شبانه افزایش یافته و به طور کلی وضعیت خواب بیمار با تکرار مداخله در هر هفته نسبت به هفته قبل بهبود یافته است (001/0>P). مواد و روش‌ها: این مطالعه، به روش تجربی از نوع کارآزمایی بالینی بر روی 80 بیمار مراجعه کننده به بخش های دیالیز بیمارستان های شهدا و شهید رحیمی خرم آباد در سال 1393 انجام گرفت. ابزار شامل پرسش نامه پیتزبرگ و فرم چارت خواب بود که به وسیله آنها، وضعیت خواب نمونه هایی که دارای مشکل خواب بودند، در شب های قبل و بعد از انجام ماساژ، مورد بررسی قرار گرفت. مداخله آموزشی در پژوهش حاضر، ماساژ پا به مدت 10 دقیقه در طول دیالیز، سه بار در هفته، برای چهار هفته متوالی بود.

Title: بررسی شکاف کیفیت خدمات فوریتهای پزشکی شهرستان خرم آباد با استفاده از مدل سروکوال

http://yafte.lums.ac.ir/article-1-1849-fa.html

مقدمه: به منظور ارائه بهتر خدمات در مراکز بهداشتی درمانی آگاهی از ادراکات و انتظارات دریافت کنندگان خدمات این مراکز و تعیین شکاف موجود بین این دو مقوله نقش بسیار مهمی را ایفاء می کند. این پژوهش با هدف ارزیابی کیفیت خدمات مرکز فوریتهای پزشکی شهرستان خرم آباد با استفاده از مدل سروکوال در سال 1391 انجام شد. بحث و نتیجه‌گیری: انتظارات مراجعین در تمام ابعاد کیفیت خدمات بالاتر از ادراکات آنان بوده است و نیاز است کیفیت ارائه خدمات در این مراکز در تمام ابعاد بویژه بعد همدلی بهبود یابد. پیشنهاد می گردد کیفیت ارائه خدمات در این مرکز و سایر مراکز بصورت دوره ای بررسی و به منظور بهبود ارائه خدمات درمانی اقدامات لازم صورت گیرد. یافته‌ها: پس از بررسیهای انجام شده تمامی ابعاد پنجگانه کیفیت خدمات ارائه شده (شامل ابعاد ملموسات، تضمین و اطمینان، پاسخگویی، مسئولیت پذیری و همدلی) شکاف منفی کیفیت وجود داشت، که بیشترین شکاف کیفیت در بعد همدلی و کمترین شکاف در ابعاد مؤلفه های ادب و اعتماد واحدهای مورد پژوهش بود. این شکاف در همه ابعاد، به استثنای بعد ادب و اعتماد از نظر آماری معنی دار بود، ولی بین سن و جنس و سطح تحصیلات مراجعین و نمره شکاف کیفیت، رابطه آماری معناداری وجود نداشت. مواد و روش‌ها: در این پژوهش توصیفی-تحلیلی400 نفر از گیرندگان خدمات فوریتهای پزشکی شهرستان خرم آباد با استفاده از روش نمونه گیری مستمر انتخاب شده و مورد بررسی قرار گرفتند. جمع آوری داده ها با استفاده از پرسشنامه استاندارد سروکوال انجام شد. تجزیه و تحلیل داده ها با استفاده از نرم افزار SPSS و به کمک آمار توصیفی و آزمون های تی زوجی، کروسکال والیس و آنووا انجام گرفت.

Title: تعیین سهم مولفه های هوش هیجانی و سبک های فرزند پروری در پیش بینی حالات روان شناختی مثبت

http://yafte.lums.ac.ir/article-1-1850-fa.html

مقدمه: از آنجایی که بنیان حالات روان شناختی مثبت به عنوان متمم رویکرد های کمبود مدار در سالهای اخیر مطرح شده است بر آن شدیم تا این شاخه جدید روان شناسی را که به مطالعه علمی نیرومندی های انسان نظر دارد بیشتر مورد توجه قرار داده و به بررسی سهم مولفه های هوش هیجانی و سبک های فرزند پروری در پیش بینی حالات روان شناختی مثبت بپردازیم. مواد و روش‌ها: در این مطالعه مقطعی 200 نفر از دانشجویان روان شناسی مقطع کارشناسی دانشگاه آزاد اسلامی واحد رودهن با استفاده از نمونه گیری خوشه ای مرحله ای انتخاب شدند. سپس با استفاده از پرسش نامه های هوش هیجانی برادبری و گریورز، سبک های فرزندپروری بامریند و پرسش نامه حالات روان شناختی مثبت رجایی و همکاران مورد ارزیابی قرار گرفتند. داده های پژوهش با استفاده ازآمار توصیفی (میانگین و انحراف استاندارد) و آمار استنباطی (رگرسیون چندگانه) به کمک نرم افزار SPSS بررسی شدند. بحث و نتیجه‌گیری: با توجه به نتایج پژوهش حاضر و اهمیت حالات روان شناختی مثبت پیشنهاد می شود که به پرورش هوش هیجانی از سنین کودکی و آموزش آن به والدین و متوجه ساختن والدین در نحوه فرزندپروری توجه بیشتری شود تا با افزایش سطح رضایتمندی افراد میزان بهداشت روانی جامعه نیز ارتقا یابد. یافته‌ها: از بین مولفه های هوش هیجانی، مولفه خودآگاهی هیجانی(464/0=β) بیشترین مقدار پیش بینی کننده را دارا بود و مدیریت رابطه پیش بینی کنندگی معنا داری را در این تحقیق نشان نمی داد. همچنین از میان سبک های فرزند پروری، سبک فرزندپروری مقتدر نیز رابطه مثبت معناداری با حالات روان شناختی مثبت داشت و نیز هیچ رابطه معناداری بین سبک فرزندپروری مستبد با حالات روان شناختی مثبت یافت نشد.

Title: بررسی مقایسه ای تأثیرآزمایشگاهی عصاره گیاه مریم گلی و کلوتریمازول بر روی کاندیداآلبیکانس جداشده از واژن زنان مبتلا به واژینیت کاندیدایی

http://yafte.lums.ac.ir/article-1-1851-fa.html

مقدمه: شایع ترین عامل واژینیت در زنان کاندیدا آلبیکانس بوده و کلوتریمازول درمان انتخابی آن می باشد. عوارض داروهای شیمیایی و مقاومت میکروارگانیسم‌ها نسبت به آنها از معضلات پزشکی بوده و استفاده از گیاهان دارویی یکی از راههای جایگزین به نظر می رسد. این مطالعه با هدف مقایسه تأثیر آزمایشگاهی عصاره گیاه مریم گلی و کلوتریمازول بر روی کاندیدا آلبیکانس جدا شده از واژن زنان مبتلا به واژینیت کاندیدائی انجام شد. بحث و نتیجه‌گیری: عصاره مریم گلی رشد کاندیدا آلبیکانس را مهار می نماید و ممکن است در درمان واژینیت ناشی از این قارچ موثر باشد. یافته‌ها: عصاره مریم گلی رشد کاندیدا آلبیکانس را مهار نمود. میانگین غلظت کلوتریمازول برای 50% MIC،µg/ml 55/0± 65/0 و برای 90% MIC، µg/ml59/3±8/4 بود. میانگین غلظت مریم گلی برای 50% MIC،mg/ml 2/24±04/24 و برای90% MIC، mg/ml4/28± 2/56 به دست آمد. مریم گلی رشد سوش استاندارد کاندیدا آلبیکانس PTCC5027را با غلظت mg/ml25/1 و mg/ml 20 به ترتیب به میزان 50% و 90% مهار نمود. مواد و روش‌ها: از100 نفر زن مبتلا به واژینیت کاندیدایی، نمونه ای از ترشحات واژن در لوله آزمایش حاوی یک سی سی سرم فیزیولوژی گرفته شد. پس از انجام مراحل کشت، 24 نمونه ایزوله کاندیدا آلبیکانس شناسایی و وارد مطالعه شد. سپس این ایزوله ها در محیط سابورو حاوی کلوتریمازول یا عصاره مریم گلی کشت و 90% و 50% MIC تعیین گردید.

Title: بررسی میزان آلودگی سالمونلایی تخم اردک و بوقلمون های مصرفی در استان فارس

http://yafte.lums.ac.ir/article-1-1852-fa.html

 مقدمه : سالمونلاها اجرام میله ای کوتاه، گرم منفی، فاقد کپسول، هوازی و بی هوازی اختیاری هستند که در انسان سبب ایجاد مسمومیت غذایی می شود. در انتقال این بیماری، مواد غذایی خام با منشاء دامی به ویژه گوشت و تخم پرندگان حائز اهمیت فراوانی هستند. با توجه به اهمیت تخم اردک و بوقلمون در انتقال سالمونلوزیس و مصرف تخم اردک و بوقلمون های محلی و غیر صنعتی در منطقه، همچنین  پی بردن به میزان آلودگی سالمونلایی مطالعه حاضر صورت پذیرفت.  مواد و روش ها: به منظور مطاله حاضر، 300عدد تخم اردک و بوقلمون های بومی جمع آوری و به آزمایشگاه منتقل گردید؛ درآزمایشگاه پس از نمونه گیری از سطح تخم های مورد برسی، پوسته آنها با اتانول80 درصد ضد عفونی و محتویات هر 5 عدد تخم اردک و بوقلمون به صورت جداگانه در یک بشر مخلوط گشته و پس از24 ساعت انکوباسیون در دمای C‏ °37 با سوآب در آبگوشت سلنیت- F تلقیح گردید. نمونه از محیط سلنیت-F به آگار سالمونلا- شیگلا منتقل شد و پس از انکوباسون در C °37  ازنظر کلنی های مشکوک به سالمونلا بررسی شدند. کلنی های مشکوک در محیط های TSI و لیزین دکربوکسیلاز تلقیح و باکتری هایی که دارای واکنش های مربوط به سالمونلا بودند بوسیله آزمایش PCR با پرایمرهای اختصاصی برای سالمونلاها و سروتیپ های سالمونلا انتریتیدیس و سالمونلا تیفی موریوم مورد بررسی قرارگرفتند.  یافته ها: از مجموع 300 تخم اردک و بوقلمون مورد بررسی پوسته 7 مورد (2/3 درصد) آلوده به سالمونلا بود. از 7 مورد آلودگی 1 مورد پوسته تخم بو قلمون  آلوده به سالمونلا تیفی موریوم بود. از طرفی 6 مورد آلودگی مربوط به پوسته تخم اردک های مورد مطالعه بود که همگی از سروتیپ سالمونلا تیفی موریوم بودند.  بحث و نتیجه گیری: با توجه به مطالعه حاضر و تحقیق سایرمحققین در مجموع می توان نتیجه گرفت میزان عفونت سالمونلا در تخم اردک و بو قلمون نسبت به تخم ماکیان کمتر بوده و به نظر می رسد انتقال عمودی سالمونلا در بوقلمون و اردک کمتر از ماکیان باشد.
